# The role of fasting on spine regeneration and bacteremia in the purple sea urchin *Strongylocentrotus purpuratus*

**DOI:** 10.1371/journal.pone.0228711

**Published:** 2020-02-13

**Authors:** David A. Scholnick, Alexandra E. Winslow

**Affiliations:** Department of Biology, Pacific University, Forest Grove, Oregon, United States of America; Chang Gung University, TAIWAN

## Abstract

Fasting has been shown to increase longevity and alter immune function in a variety of animals, but little is understood about how reduced caloric intake may impact regeneration and infections in animals that must regularly repair and regenerate tissue in marine environments that contain high levels of bacteria. We examined the possibility that fasting could enhance spine regeneration and reduce bacteremia in the purple sea urchin *Strongylocentrotus purpuratus*. A small number of spines were removed from urchins and rates of spine regrowth and levels of culturable bacteria from the coelomic fluid were measured for 21 days in fed and fasted urchins. Fasted urchins had higher rates of spine regrowth and lower levels of colony-forming units (CFU) per milliliter of coeolomic fluid. The predominant bacteria in the coelomic fluid was isolated and identified by DNA sequence-based methods as *Vibrio cyclitrophicus*. After 21 days, fasted and fed urchins were injected with *V*. *cyclitrophicus*. Two hours after injection, fed urchins had about 25% more culturable bacteria remaining in their coelomic fluid compared to fasted urchins. We found no evidence that fasting altered coelomic fluid cell number or righting response, indicators of physiologic and behavioral stress in urchins. Our results demonstrate that *V*. *cyclitrophicus* is present in purple urchin coelomic fluid, that fasting can increase spine regeneration and that fasted urchins have much lower levels of culturable bacteria in their coelomic fluid than fed urchins. Overall, our data suggests that fasting may ultimately reduce bacteremia and infection in injured or damaged urchins.

## Introduction

Dietary restrictions, such as fasting, have been linked to numerous health benefits including increased life span, protection against age-related pathologies, and changes in immune function in both vertebrates and invertebrates [1–4]. Although immune defense is costly, immune functions can be enhanced by food limitations [5,6]. Sea urchins appear to be particularly well adapted to intermittent fasting. For example, the purple sea urchin, *Strongylocentrotus purpuratus*, regularly faces food limitations due to seasonally changes in kelp availability and loss of kelp due to large numbers of active urchin foragers [7]. Sea urchins are also known to limit foraging when damaged or in the presence of predators [8]. During caloric restrictions, urchins are able to maintain metabolism and growth through release of lipids and carbohydrates via somatic and gonadal tissues when food is not available [9–11].

Tissue damage and spine loss are associated with wave shock, debris, predation, disease and limited caloric intake in sea urchins [12,13]. As such, spine regeneration and wound-healing are important factors for urchin success. Spine regeneration appears to be critical for locomotion, feeding, and defense against predators and has important impacts on survival and reproductive success [14]. Spine regeneration involves healing and repair of the epidermis, followed by biomineralization of new spines potentially involving coelomocytes acting as stem cells [14]. In *Strongylocentrotus purpuratus*, spine regeneration is rapid with complete regeneration in several months depending upon temperature [15,16]. Urchins are even able to maintain rapid rates of spine regeneration during stressful conditions such as prolonged exposure to elevated pCO_2_ although new spine integrity in elevate CO_2_ conditions appears to be reduced [17]. One might expect that reduced caloric intake could potentially negatively impact spine regenerations and elevate risks of bacterial infections.

Healthy urchins are able to rapidly remove bacteria from coelomic fluid. After bacterial injection, 90–99% of injected bacteria are cleared from coelomic fluid of *S*. *purpuratus* within the first 3–6 hours corresponding to reductions in coelomocyte counts [18]. Previous studies have reported that sea urchin immune cell concentrations and composition change in response to environmental factors and stress [19,20]. Matranga et al. [19] found elevated red spherula counts in *Paracentrotus lividus* urchins with spineless lesions covering 10 to 20% of the body whereas mechanical damage is known to increase ceolomocyte number and humoral molecule expression [20]. While many studies have investigated the immune response in sea urchins [21–23], few have considered the impact of spine damage on immune function and bacteremia.

In the current investigation, we examined the effect of 21 days of fasting on spine regeneration rates and bacteremia in the purple sea urchin *S*. *purpuratus*. We hypothesized that limited food availability could potentially disrupt spine regeneration and immune function associated with tissue repair and pathogen exposure. We measured the effect of fasting for 21 days on spine regeneration rates, bacteremia, and coelomocyte concentrations in urchin that had undergone spine damage. Following the fast, differences in bacterial clearance rates, coelomocyte cell counts and righting response were measured in both fasted and fed animals 2 h post bacterial injection. Given the beneficial effects of dietary restrictions previously reported in other animals [3] our working hypothesis was that short-term caloric-restrictions had the potential to alter tissue regeneration and wound healing.

## Materials and methods

### Animal care

Purple sea urchins, *Strongylocentrotus purpuratus* were purchased from Marinus Scientific, Newport Beach, CA (mean mass = 50.3 g ± 3.3; mean test diameter = 52.2 mm ± 1.0). Urchins were kept in UV-sterilized recirculating seawater at 12°C and 32 ppt salinity. All urchins were fed and acclimated for two weeks before beginning experiments. Sea urchins were fed 2 X 2 cm pieces of *Macrocystis pyrifera* every other day. During feeding, all remaining kelp was removed and cages resupplied with new kelp. In most cases no food remained in the cages after 12 h.

### Spine regeneration

Five spines were amputated from one ambulacral section at the base of the spine and five spines were amputated from the opposite ambulacral section approximately in the middle of the spine. Spines were amputated using sterile surgical scissors under a dissecting microscope. Spine regeneration rates were determined by measuring spine length relative to initial via digital image analysis (ImageJ software). Individual spines could be tracked over time given the relative position along the ambulacral section. Following spine removal, all urchins were housed individually in rectangular mesh cages (15 X 25 cm) constructed from plastic hardware cloth (1.27 cm mesh) held together with cable ties. All protocols were performed twice on two separate groups of urchins.

### Plating

The number of culturable bacteria in coelomic fluid was measured as colony forming units (CFU) per ml coelomic fluid by removing aliquots of sterile coelomic fluid and transferring it to tryptic soy agar (TSA) supplemented with 3.5% NaCl. Three sterile glass beads were used to spread the coelomic fluid across agar plates before plates were incubated at 12°C for 48 hours. The number of bacterial colonies were counted under a dissecting microscope and recorded for each plate to quantify the number of culturable bacteria in the coelomic fluid of each urchin at each of the time points tested. Water samples were plated in a similar fashion at each time point to ensure that culturable bacteria levels were not present in the water in which the urchins were held.

### Bacterial challenge

A single bacterial colony type was identified by morphological characteristics as the most common and persistent coelomic fluid bacterium (>95% of CFU). Isolates were identified as *Vibrio cyclitrophicus* by using the first 500 base pairs on the 16S ribosomal gene and universal primers (5’-AGAGTTTGATCCTGGCTCAG and 5’-TTACCGCTGCTGGCA). Identification was confirmed by two independent labs: The Clinical Microbiology Laboratory at the University of Washington Medical Center and Charles River Microbial Solutions. Isolated *V*. *cyclitrophicus* was used for subsequent bacterial injection experiments.

Following 21 days of fasting, urchins were injected with live *Vibrio cyclitrophicus* mixed in sterile seawater. Prior to injection, *V*. *cyclitrophicus* was streaked onto a TSA plate supplemented with 3.5% NaCl and kept for 48 hours at 12°C. The concentration of *V*. *cyclitrophicus* was determined spectrophotometrically at 540 nm and diluted with sterile seawater to obtain an injection dose of approximately 10,000 CFU ml^-1^ (coelomic fluid volume estimated from [18]). The bacterial dose of an additional 10,000 culturable bacteria ml^-1^ was selected to profile a successful defense against a persistent bacterial pathogen (a reduction of injected CFU of over 50% over 2 h). The bacterial suspension (5.5 μl g^-1^ body weight) was injected through the peristomial membrane and the animals inverted several times to aid in mixing the coelomic fluid. After 2 hours, about 250 ul of coelomic fluid was removed and aliquots plated on TSA with 3.5% NaCl. Plates were incubated at 12°C for 96 h, and the number of bacterial colonies was counted under a dissecting microscope to determine CFU ml^-1^.

To determine the influence of fasting and bacterial injection on number of circulating coelomocytes, a separate coelomic fluid samples (<100 μl) was removed from the peristomial membrane and immediately mixed with cold 20% neutral buffered formalin. Cells from four different aliquots were counted in a hemocytometer and averaged.

### Behavioral response

Righting response time was measured initially, 21 days after spine removal and 2 h post injection for both fed and fasted animals. Amount of time taken to return to a normal oral surface down positon following inversion was measured in a separate glass aquarium, filled with sterile well aerated seawater at 12°C.

### Statistical analysis

To determine whether fasting influenced CFU, coelomocyte cell numbers, or rate of righting, a two-way RM ANOVA was performed over time for fasted and fed animals. If statistical differences between treatments were found, *post hoc* comparisons between treatments were performed using the Holm-Sidak method. The effect of fasting on CFU, coelomocyte number and rate of righting was analyzed by unpaired t-test. All data are reported as mean ± standard error unless noted otherwise. Statistical analyses were done using SigmaStat 3.5 (Systat Software Inc., San Jose, CA).

## Results

### Spine regeneration

**Fig 1:**
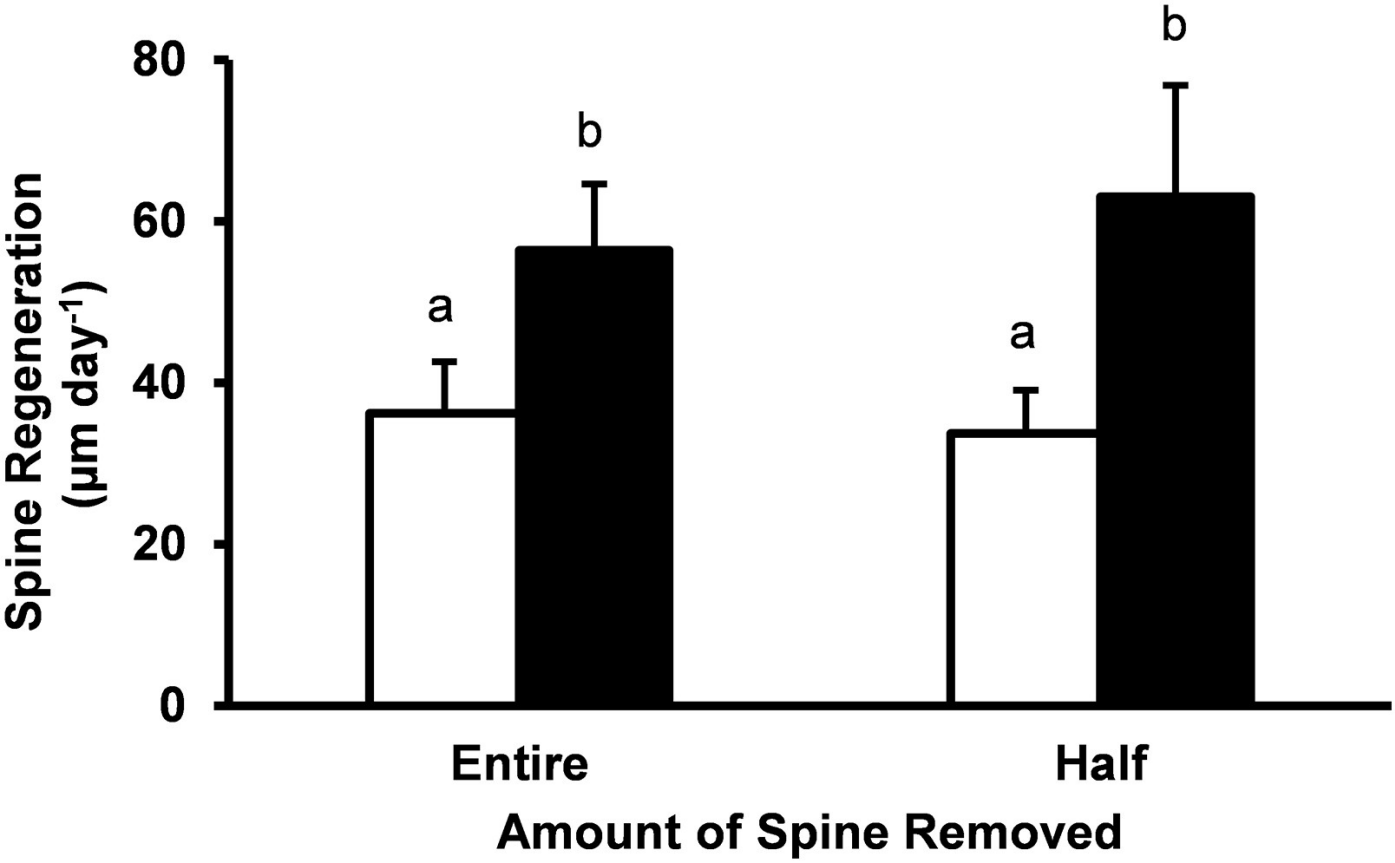
Spine regeneration rate. Rate of spine regeneration (μm day^-1^) for spines cut at the base (entire) or at half the spine length (half) after 21 days of feeding ad libitum (white bars) or fasted (black bars). Mean values ± standard error are shown (n = 9 for each treatment group). Fed animals had significantly lower spine regeneration rates for whole and half spine removal (*P* = 0.0421, t = 2.772; *P* = 0.00752, t = 3.0871 respectively).

Fasted *Stronglyocentrotus purpuratus* had significantly increased rates of spine regeneration compared to fed urchins for both whole and half amputated spines (Fig 1; P < 0.05). Rate of regeneration was almost double for fasted urchins (61 μm day^-1^ of regrowth) compared with fed animals (35 μm day^-1^ of regrowth). Rate of spine regeneration was not influenced by the length of the spine removed, entire spine or half spine amputations had similar regeneration rates within treatment groups (P > 0.05).

### Bacteremia

**Fig 2:**
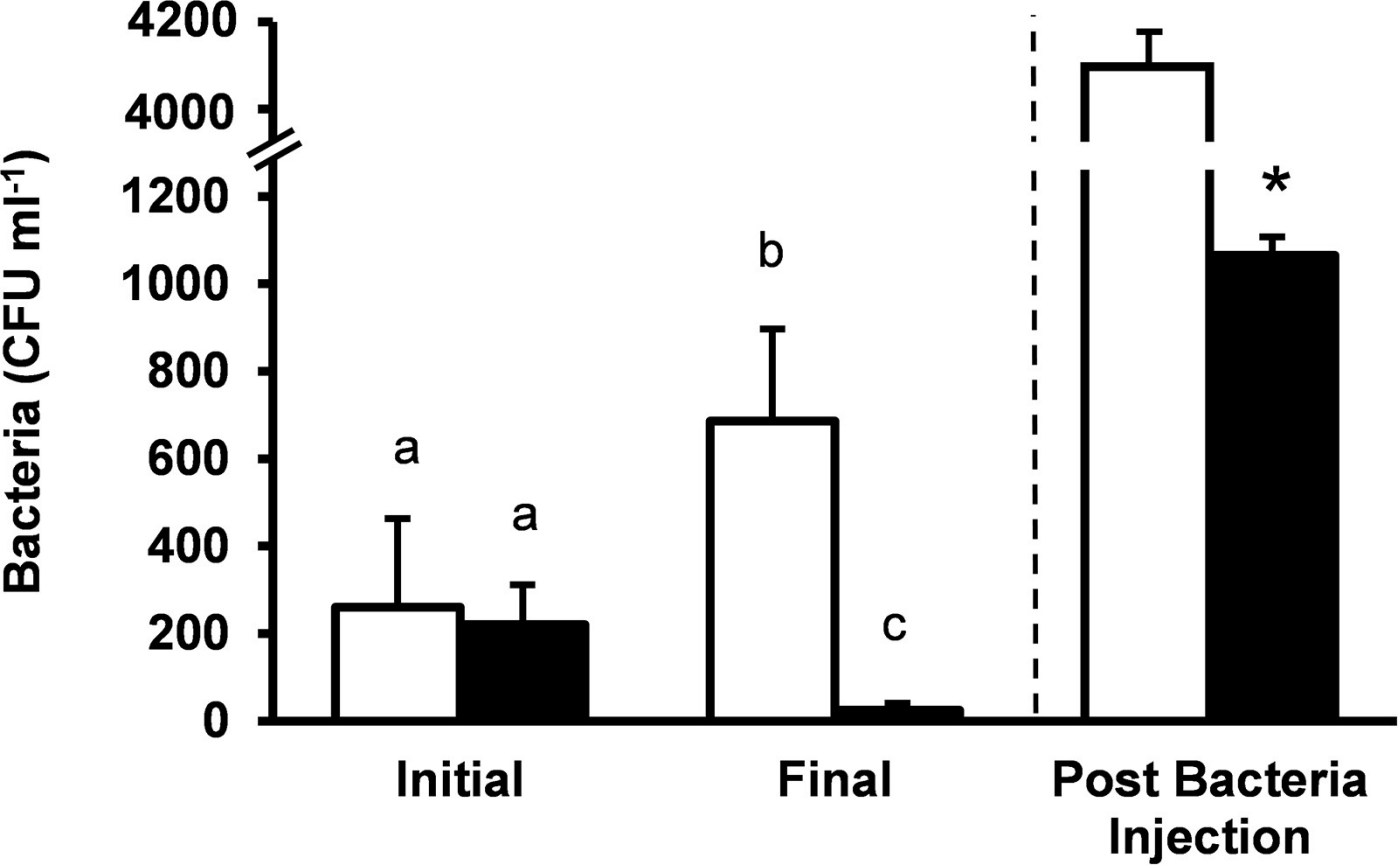
Fasting decreases colony forming units. Coelomic fluid bacteria measured as the total number of colony-forming units (CFU) per milliliter of coelomic fluid. Bacteremia was assessed initially and 21 days after spine amputation in animals that were either fed (white bars) or fasted (black bars). Following fasting, urchins were injected with *Vibrio* and total bacterial CFU per milliliter of coelomic fluid were counted 2 hours post injection. Mean values ± standard error are shown (n = 9 for each treatment group). Significant differences between groups and over time are indicated by letters above bars (two-way ANOVA, *P* < 0.05). Following bacterial injection, the net increase in culturable bacteria in coelomic fluid was significantly higher in urchins that had been fed for 21 days compared to fasted urchins 2 h post injection (*P* = 0.032, t = 2.3594, unpaired t-test).

Following spine removal, fasted *Stronglyocentrotus purpuratus* had significantly lower levels of pre-existing culturable bacteria in the coelomic fluid when compared to fed urchins (Fig 2; *P* = 0.032, *n* = 9 for each treatment). Thus, after 21 days, fed urchins had almost 30 times more culturable bacteria per milliliter of coelomic fluid compared to fasted urchins (about 683 compared to 21 CFU ml^-1^ respectively). Initial levels of culturable bacteria in the coelomic fluid were low and remained stable at about 246 ± 76.3 CFU ml^-1^ for all urchins during the two week acclimation period prior to spine removal.

The ability of 21 day fasted *Strongylocentrotus purpuratus* to remove injected *Vibrio cyclitrophicus* from the coelomic fluid was measured 2 h post injection. The remaining number of culturable bacteria after 2 h was significantly larger in fed urchins than fasted urchins (*P* = 0.032; Fig 2). Of the 10,000 CFU ml^-1^ that were injected into the coelomic fluid at the end of the treatment, only 13% remained in the fasted urchins (about 1,020 of the injected CFU ml^-1^ remained) compared to 40% remaining in the fed animals (about 3,750 ml^-1^ of the injected CFU ml^-1^).

### Coelomocytes

**Fig 3:**
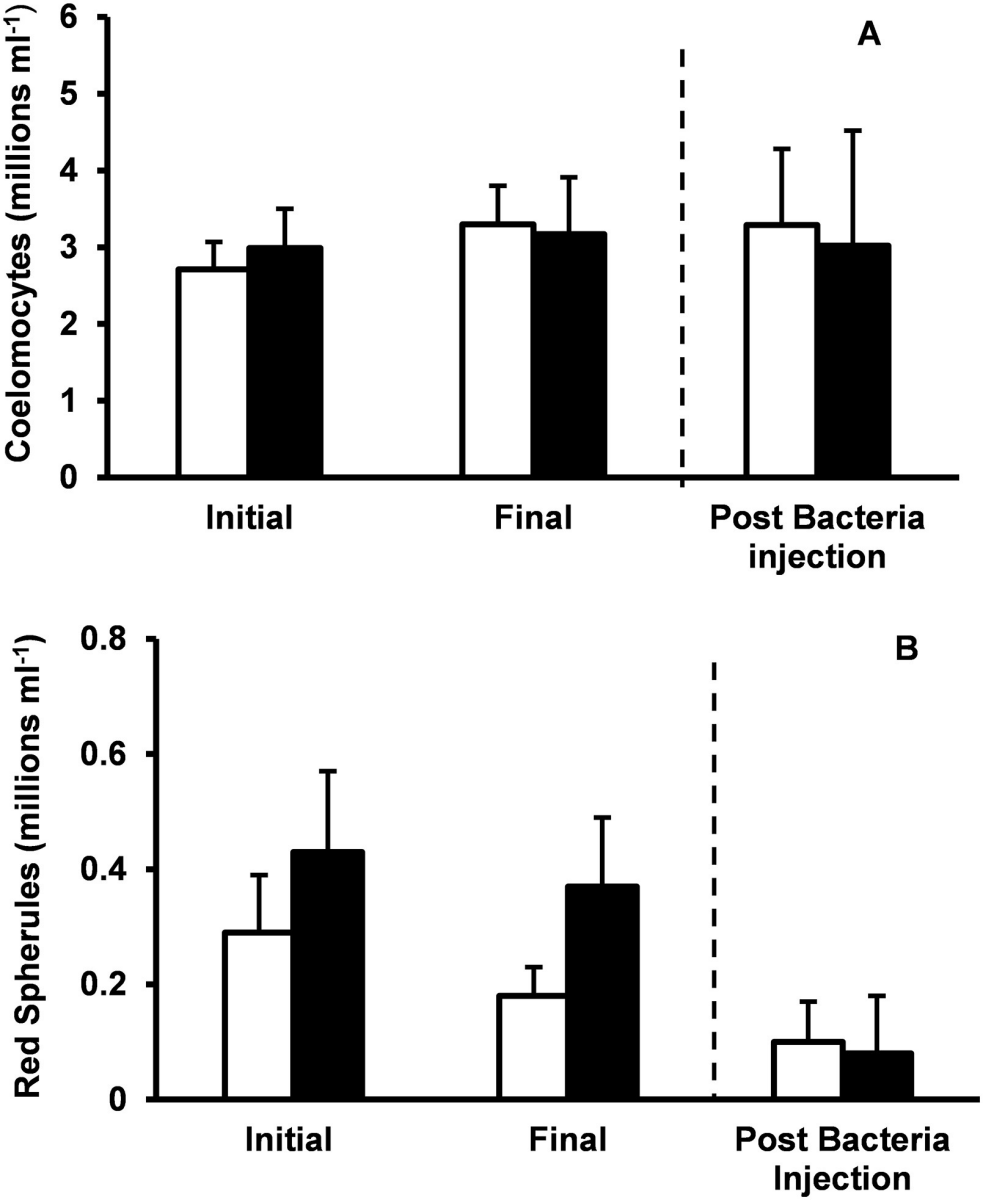
Change in coelomocytes. Total number of coelomocytes (A) and red spherules (B) per milliliter of urchin coelomic fluid in fed (white bars) and fasted (black bars) urchins. Cells were counted initially and 21 days after spine amputation. Following fasting, urchins were injected with *Vibrio* and coelomic cells counted 2 hours post injection. Mean values ± standard error are shown (n = 9 for each treatment group). Fasting had no significant impact on coelomocyte number compared to animals that were fed (*P* > 0.05, RMANOVA). Decreases in coelomic cell numbers following injection of bacteria did not differ between fed and fasted urchins (*P* > 0.05, unpaired t-test).

The total number of circulating coelomocytes and red spherules was unaltered by spine removal or fasting (Fig 3; RM ANOVA, P > 0.05). Changes in total number of coelomocytes and number of red spherules between fed and fasted animals was measured 2 h after injection of *Vibrio cyclitrophicus* into the coelomic fluid. The total number of coelomocytes and total number of red spherules were similar in fed and fasted urchins following bacterial injection (P > 0.05).

### Behavior and size

**Fig 4:**
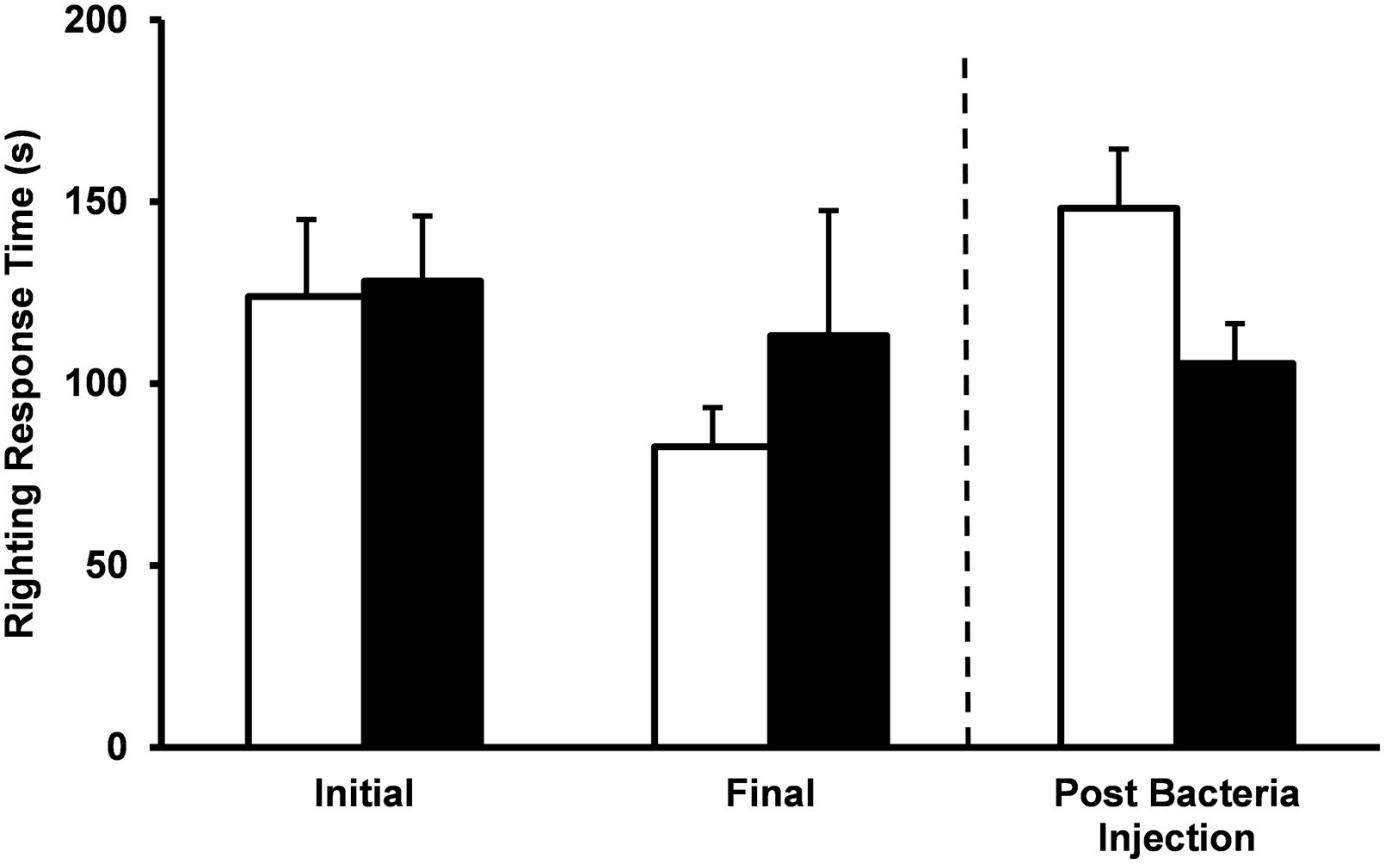
Righting response. Righting time, the time taken to return to upright position following inversion, in fed (white bars) and fasted (black bars) urchins. Righting time was measured initially and 21 days after spine amputation. Following fasting, urchins were injected with *Vibrio* and righting time measured 2 hours post injection. Mean values ± standard error are shown (n = 9 for each treatment group). Fasting had no significant impact on righting time compared to fed animals (*P* > 0.05, ANOVA). There was no difference in righting response time following injection of bacteria between fed and fasted urchins (*P* > 0.05, unpaired t-test).

Righting response time was not affected by spine removal, fasting or bacterial injection (Fig 4; P >0.05). In addition, there was no significant change in total mass or test diameter from initial measurements and final measurements after 28 days of fasting.

## Discussion

Our results show that fasting accelerated spine regeneration in *Strongylocentrotus purpuratus*. Rates of spine regeneration were over 1.5 times great in urchins fasted for 21 days compared to fed urchins (Fig 1). Fasting has previously been reported to optimize cellular energy metabolism and stress resistance pathways in a large variety of eukaryotes [1]. For example, mice that underwent long term dietary restriction have been shown to exhibit enhanced wound healing [24]. In *S*. *purpuratus*, spine regeneration appears to negatively impact gonadal growth thereby potentially impacting reproductive potential [11]. The results of the current experiment suggest that caloric restriction in sea urchins has the ability to upregulate metabolic processes associated with spine regeneration, similar to a large variety of phyla where fasting has been shown to provide metabolic benefits to improve health and lifespan [3]. While the cellular and molecular pathways associated with fasting in invertebrates are not well characterized, there is evidence that stem cells may play a role in tissue regeneration in sea urchins [25].

We identified *Vibrio cyclitrophicus* in the coelomic fluid of *Strongylocentrotus purpuratus*. *V*. *cyclitrophicus* is a marine bacterium known to degrade polycyclic aromatic hydrocarbons and has been previously isolated from eel, clams, and sea cucumber [26–28]. Although the pathophysiology of *V*. *cyclitrophicus* in sea urchins remains unclear, *V*. *cyclitrophicus* has been associates with skin ulceration syndrome and visceral rejection syndrome in cultured sea cucumbers [28]. Two hours after injection of *V*. *cyclitrophicus*, culturable bacteria remain in the coelomic fluid of both fed and fasted *S*. *purpuratus* (Fig 2). These results are consistent with previous measurements showing significant amounts of bacteria remained in the coelomic fluid of *S*. *purpuratus* 6 h after injection of both Gram negative and Gram positive bacteria [18]. In the current study, fasted *S*. *purpuratus* were able to remove about 25% more injected bacteria from the coelomic fluid 2 h post-injection than fed urchins (Fig 2). The results from the current study suggest that fasting may enhance bacterial clearance and that fasting could be beneficial in preventing bacterial infections following tissue damage and subsequent regeneration. Previous studies have demonstrated that illness-induced anorexia can enhance immune function and recovery in invertebrates [5,6,29].

Although levels of coelomic CFU in fed urchins were significantly higher than in fasted urchins 21 days after spine removal, we found no differences in total ceolomocytes or red spherules between fed and fasted urchins (Fig 3). Previous studies have reported that the number of coelomocytes present in the perivisceral fluid and the expression of humoral molecule following mechanical injury increase due to mechanical damage [20]. In addition, elevated red spherule numbers in the perivisceral fluid are associated with lesions and lack of spines [19]. Caloric restriction has been shown to delay immunosenescence in animals and can potentially improve immune response, although there is some evidence that caloric restriction can limit host defense against pathogenic infections [reviewed by 30]. Further examination of the effects of fasting on coelomocyte function, immune gene expression and antimicrobial proteins in the coelomic fluid appears warranted.

Previous studies have reported that reduced caloric intake and decreased diet quality have been linked to spine shedding and elevated righting times [12,13]. In the present study we found that 21 days of fasting had no impact on righting (Fig 4), although, righting response has been used as an indicator of stress and physiologic fitness [17,31,32]. Haag et al., [11] found that damaged *S*. *purpuratus* will remain in pits thereby limiting ability to feed, supporting our finding that fasting may be beneficial for wound repair and spine regeneration. We observed little to no spine loss over the 21 days urchins were in mesh cages for either in fed or fasted animals.

Overall, our results suggest that caloric restrictions can alter the ability of sea urchins to regenerate tissue and influence susceptibility to infectious disease. Our results support the hypothesis that dietary restrictions can provide protection from bacteremia and that short-term fasting may have important implications for wound healing and tissue regeneration in echinoderms. We report that 21 days of fasting had little impact on coelomocyte numbers or righting behavior which have previously been used as indicators of stress in urchins [16,19]. Physiological changes that occur with caloric restrictions may be important mechanisms for regeneration and infection in sea urchins where tissue damage, spine loss, and regeneration are important functions for survival.

## Supporting information

S1 Data (XLSX)
